# Immunogenicity and duration of antibodies after vaccination with a two-dose series of the nine-valent human papillomavirus vaccine among Alaska Native children: a prospective cohort study

**DOI:** 10.1186/s12879-025-10961-z

**Published:** 2025-04-30

**Authors:** Jonathan Steinberg, Gitika Panicker, Elizabeth R. Unger, Ian Blake, Rayleen M. Lewis, Jesse Geis, Dana Bruden, Marc Fischer, Lauri E. Markowitz, Michael G. Bruce

**Affiliations:** 1 Arctic Investigations Program, Division of Infectious Disease Readiness and Innovation, National Center for Emerging and Zoonotic Infectious Diseases, Centers for Disease Control and Prevention, Anchorage, Alaska, U.S.A.; 2 Division of High-Consequence Pathogens and Pathology, National Center for Emerging and Zoonotic Infectious Diseases, Centers for Disease Control and Prevention, Atlanta, GA U.S.A.; 3 Division of Viral Diseases, National Center for Immunization and Respiratory Diseases, Centers for Disease Control and Prevention, Atlanta, GA U.S.A.

**Keywords:** Human papillomavirus viruses, Vaccines, Alaska

## Abstract

**Background:**

Human papillomavirus (HPV)-associated cancers are vaccine preventable. In 2016, the previously recommended three-dose HPV vaccination series was changed to a two-dose series and nine-valent HPV vaccine (9vHPV) became the only HPV vaccine available in the United States. Data on longer-term duration of antibodies following a 9vHPV two-dose series are limited. We evaluated the immunogenicity and duration of antibodies up to three years after vaccination with a two-dose series of 9vHPV in a cohort of Alaska Native children.

**Methods:**

We enrolled Alaska Native children aged 9–14 years who received 9vHPV in Anchorage, Alaska during 2017–2018. We collected sera at six months after dose one and at one month, one year, and three years after dose two to measure type-specific immunoglobulin G (IgG) concentrations for the 9vHPV types (HPV6/11/16/18/31/33/45/52/58). Aggregate type-specific IgG concentrations were reported as geometric mean concentrations (GMC).

**Results:**

A total of 227 children completed the two-dose series of 9vHPV and provided ≥ 1 blood sample. The median age at enrollment was 11.0 years (range: 9.0–14.6) and was similar between males and females (*p* = 0.11). At one month after dose two, all 197 participants with available serum were seropositive for all 9vHPV types. Among 145 participants who had a specimen available at three years after dose two, 134 (92%) remained seropositive for all 9vHPV types. GMC peaked for all types at one month post dose two and remained higher at three years post dose two compared to six months post dose one.

**Conclusions:**

We found high immunogenicity and antibody persistence after a two-dose series of 9vHPV in this cohort of Alaska Native children. Further follow-up will determine duration of antibody detection in this cohort.

**Supplementary Information:**

The online version contains supplementary material available at 10.1186/s12879-025-10961-z.

## Introduction

Persistent infection with human papillomavirus (HPV) can cause cervical and other anogenital and oropharyngeal cancers [1, 2]. More than 36,000 cases of HPV-attributable cancer are diagnosed in the United States each year [3].

In 2006, the Advisory Committee on Immunization Practices (ACIP) recommended a three-dose series of quadrivalent HPV vaccine (4vHPV) for girls aged 11–12 years in the United States; this recommendation was later expanded to include boys [4, 5]. 4vHPV protects against two oncogenic types that cause most HPV-attributable cancers (HPV 16 and 18) and two types that cause anogenital warts (HPV 6 and 11) [4]. In 2015, nine-valent human papillomavirus vaccine (9vHPV) was recommended for use in the United States [6]. 9vHPV is safe and effective and protects against the four types in 4vHPV, plus five additional oncogenic types (HPV 31, 33, 45, 52, 58) [6]. In 2016, ACIP recommended a two-dose series of HPV vaccine for U.S. children aged 9–14 years [7].

No waning of protection has been detected through 10 years following vaccination with a three-dose series of 4vHPV or 9vHPV [5, 8, 9]. However, data on longer-term duration of antibodies following receipt of a two-dose series of 9vHPV are limited [10–12]. Historically, Alaska Native peoples were at increased risk for HPV-associated cancers [13, 14]. Alaska Native and other Indigenous peoples also have been underrepresented in vaccine research [15, 16]. We evaluated the immunogenicity and duration of antibodies up to three years after vaccination with a two-dose series of 9vHPV in a cohort of Alaska Native children.

## Methods

### Study design and sample size

This is a post-licensure, prospective cohort study to evaluate the immunogenicity of a two-dose series of 9vHPV among Alaska Native children. We aimed to recruit 250 children (130 male and 120 female) with the intent to create a cohort of individuals who will be followed for 20 years following vaccination with 9vHPV. Based on our experience with other long-term cohorts of vaccine recipients in Alaska, we expect to retain approximately 40% of enrollees through at least 15 years post vaccination.

### Study participants

We enrolled Alaska Native children aged 9–14 years who presented to the pediatric and primary care clinics on the Alaska Native Health Campus in Anchorage, Alaska during October 2017–August 2018. Study personnel recruited participants during routine medical visits and administered a brief questionnaire (Supplemental file) and reviewed medical records to determine eligibility for enrollment. Children were considered ineligible to participate in the study if they: 1) previously received any HPV vaccine; 2) had a hypersensitivity to any component of 9vHPV; 3) had a medical condition that reduces cell-mediated or humoral immunity; 4) received immunomodulatory therapy within the previous six months; or 5) were pregnant. Prior receipt of HPV vaccine was assessed with the Alaska immunization registry, medical record review, and a parental questionnaire. All vaccine providers in the state of Alaska are required by law to report vaccinations to the immunization registry, with no provision for parents or children to opt out of the reporting requirement [17].

### Study procedures and data collection

Two doses of 9vHPV were administered to participants 5–12 months apart according to ACIP recommendations [7]. Approximately 5 mL of blood was collected by venipuncture from each participant at up to four time points: immediately prior to administration of the second dose of 9vHPV, one month after the second dose, one year after the second dose, and three years after the second dose. Prior to collection of each blood sample, study personnel reviewed participants’ medical records and the Alaska immunization registry to confirm continued eligibility for participation (i.e., residence in the Anchorage area and no receipt of additional doses of HPV vaccine).

### Laboratory testing

Blood samples were centrifuged and sera was drawn off into 0.5 mL aliquots, which were frozen at -20 °C and sent to the Centers for Disease Control and Prevention (CDC) HPV laboratory in Atlanta, G.A. for testing. We used a multiplex virus-like particle enzyme-linked immunosorbent assay to measure type-specific immunoglobulin G (IgG) antibody concentrations for the 9vHPV types, as previously described [18]. Cutoff values were determined using 200 anonymized sera from children as negative controls. Seropositivity in international units per milliliter (IU/mL) was defined as ≥ 7.6 for anti-HPV 6, ≥ 5.2 for anti-HPV 11, ≥ 1.6 for HPV 16, ≥ 3.5 for anti-HPV 18, ≥ 5.5 for anti-HPV 31, ≥ 6.8 for anti-HPV 33, ≥ 4.2 for anti-HPV 45, ≥ 6.2 for anti-HPV 52, and ≥ 12.8 for anti-HPV 58.

### Statistical analysis

All statistical analyses were conducted using SAS version 9.4 (SAS Institute, Cary, N.C). Two-sided *p*-values < 0.05 were considered statistically significant. Categorical variables were compared using the likelihood ratio chi-square test. Continuous variables reported as medians were compared with the Wilcoxon rank-sum test.

Immunogenicity data were analyzed for participants who completed the two-dose series of 9vHPV and provided at least one post-vaccination blood sample. Individual antibody concentrations below the laboratory assay’s lower limit of quantitation were assigned a value halfway between zero and the lower limit. We estimated 95% confidence intervals (CI) for seropositivity proportions using the Clopper-Pearson exact binomial method. Antibody concentrations were reported as geometric mean concentrations (GMC). GMC at three years post vaccination were stratified by sex and age at enrollment, with age modeled as a two-level categorical variable (ages 9–11 years and ages 12–14 years). We used independent t-tests assuming unequal variances to compare GMC for each HPV type at three years post vaccination by sex; we did not conduct formal hypothesis tests comparing GMC by age group due to the small number of participants aged 12–14 years at enrollment. Multiple linear regression was used to control for the effect of age at enrollment on the association between sex and antibody duration at three years post vaccination. In these regression models, log-transformed, type-specific, three-year antibody GMC was the response variable and sex and age were the explanatory variables.

## Results

**Fig. 1:**
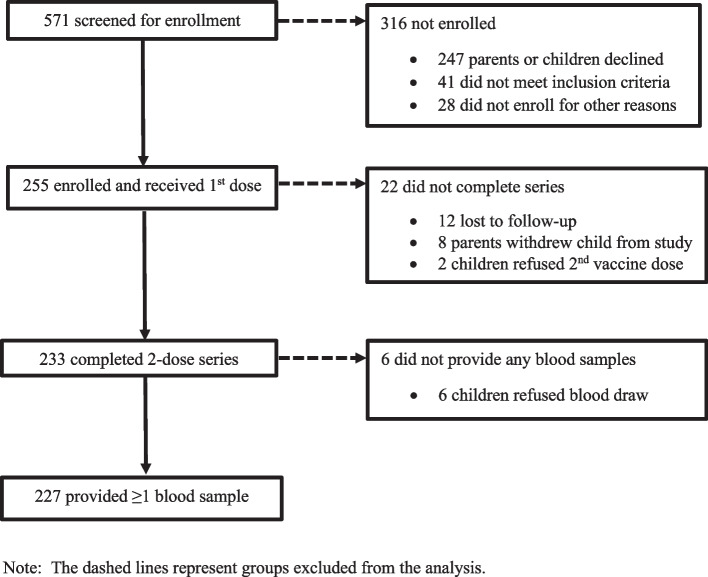
Flow diagram for participants in a study of immunogenicity of a two-dose series of nine-valent human papillomavirus vaccine among Alaska Native children

Of the 571 children screened for enrollment, 255 (45%) were enrolled and received the first dose of 9vHPV (Fig. 1). Among those enrolled, 227 (89%) completed the two-dose series and provided at least one post-vaccination blood sample. For these 227 children included in the analysis, median age at enrollment was 11.0 years (range: 9.0–14.6) and was similar between males (10.7 years) and females (11.1 years) (*p* = 0.11). Overall, 185 (81%) participants were aged 9‒11 years (Table 1). Most participants (223/227, 98%) received the second dose within 5–12 months after the first dose (median: 6.0 months; interquartile range (IQR): 6.0–7.0 months); 4 (2%) children received the second dose 13‒14 months after the first dose. Excluding the 4 children who received the second dose > 12 months after the first dose, the mean time between doses was 6.6 months.

**Table 1:** Demographic characteristics of Alaska Native children who completed a two-dose series of nine-valent human papillomavirus vaccine and provided ≥ 1 serum sample

	**All participants**		**Male**		**Female**		***p*-value**
	**[*N*** **= 227]**		**[*N*** **= 122]**		**[*N*** **= 105]**		
	**No**	**(%)**	**No**	**(%)**	**No**	**(%)**	
Age at enrollment, years							0.16
9	57	(25)	34	(28)	23	(22)	
10	54	(24)	32	(26)	22	(21)	
11	74	(33)	36	(30)	38	(36)	
12	27	(12)	12	(10)	15	(14)	
13	11	(5)	4	(3)	7	(7)	
14	4	(2)	4	(3)	0	(0)	

Overall, 205 (90%) children provided a blood sample at six months after dose one, 197 (87%) at one month after dose two, 172 (76%) at one year, and 145 (64%) at three years; 107 (47%) participants provided a serum sample at all four time points. The median number of days between vaccination and post-dose one sample collection was 196 days (range: 149–388 days; IQR: 184–225). For sample collections after series completion, the median time since dose two was 35 days (range: 25–118 days; IQR: 32–46) for the one-month sample, 365 days (range: 300–466 days; IQR: 355–382) for the one-year sample, and 1,097 days (range: 1,065–1,186 days; IQR: 1,083–1,132) for the three-year sample. Excluding the 4 children who received the second dose > 12 months after the first dose, the mean time between vaccination and post-dose 1 sample collection was 213 days; the mean time between dose 2 and sample collection was 40 days for the one-month sample, 371 days for the one-year sample, and 1,109 days for the three-year sample.

Following receipt of one dose of 9vHPV, 85% of the 205 participants were seropositive for HPV 45, 94% for HPV 18, 98% for HPV 33, and ≥ 99% for the remaining six HPV types (Table 2). At one month post dose two, all of the 197 participants with available serum were seropositive for all 9vHPV types. At one year post dose two, all of the 172 participants with available data remained seropositive against four of the 9vHPV types (HPV 6, 11, 16, 58) and ≥ 95% were seropositive for the other five types (HPV 18, 31, 33, 45, 52). At three years post dose two, all 145 participants with data were seropositive against six 9vHPV types (HPV 6, 11, 16, 33, 52, 58) and ≥ 92% remained seropositive against the other three types (HPV 18, 31, 45). Among the 127 participants with data available at one year and three years post dose two, 5 (4%) were seronegative for ≥ 1 HPV type at one year post dose two and seropositive for the same type at three years. These five participants were aged 14–17 years at the three-year time point.

**Table 2:** Seropositivity and 95% confidence intervals (CI) among Alaska Native children after receipt of nine-valent human papillomavirus (HPV) vaccine

	**6 months** **after dose 1** **[*N*** **= 205]**			**1 month** **after dose 2** **[*N*** **= 197]**			**1 year** **after dose 2** **[*N*** **= 172]**			**3 years** **after dose 2** **[*N*** **= 145]**		
**Type**	**No**	**(%)**	**95% CI**	**No**	**(%)**	**95% CI**	**No**	**(%)**	**95% CI**	**No**	**(%)**	**95% CI**
HPV 6	203	(99)	(97–100)	197	(100)	(98–100)	172	(100)	(98–100)	145	(100)	(97–100)
HPV 11	202	(99)	(96–100)	197	(100)	(98–100)	172	(100)	(98–100)	145	(100)	(97–100)
HPV 16	204	(> 99)	(97–100)	197	(100)	(98–100)	172	(100)	(98–100)	145	(100)	(97–100)
HPV 18	193	(94)	(90–97)	197	(100)	(98–100)	165	(96)	(92–98)	139	(96)	(91–98)
HPV 31	202	(99)	(96–100)	197	(100)	(98–100)	169	(98)	(95–100)	144	(99)	(96–100)
HPV 33	201	(98)	(95–99)	197	(100)	(98–100)	171	(99)	(97–100)	145	(100)	(97–100)
HPV 45	174	(85)	(79–89)	197	(100)	(98–100)	164	(95)	(91–98)	134	(92)	(87–96)
HPV 52	203	(99)	(97–100)	197	(100)	(98–100)	170	(99)	(96–100)	145	(100)	(97–100)
HPV 58	203	(99)	(97–100)	197	(100)	(98–100)	172	(100)	(98–100)	145	(100)	(97–100)

For all 9vHPV types, GMC peaked at one month post dose two and remained higher at three years compared to GMC six months post dose one (Table 3). From one month to one year post dose two, GMC declined by a median of 89% (range: 88‒90%) for the 9vHPV types. From one year to three years post dose two, GMC declined by a median of 53% (range: 44‒57%).

**Table 3:** Geometric mean concentrations (GMC) and 95% confidence intervals (CI) for type-specific human papillomavirus (HPV) immunoglobulin G antibodies following receipt of nine-valent HPV vaccine among Alaska Native children

Type	6 months after dose 1 [*N* = 205]		1 month after dose 2 [*N* = 197]		1 year after dose 2 [*N* = 172]		3 years after dose 2 [*N* = 145]	
	GMC^a^	(95% CI)	GMC^a^	(95% CI)	GMC^a^	(95% CI)	GMC^a^	(95% CI)
HPV 6	59	(52–67)	4,535	(4,067–5,056)	469	(396–555)	220	(183–263)
HPV 11	42	(37–48)	3,727	(3,390–4,097)	374	(324–432)	171	(146–200)
HPV 16	22	(20–25)	1,781	(1,610–1,969)	219	(188–254)	94	(80–111)
HPV 18	13	(12–15)	635	(566–713)	62	(52–76)	27	(23–32)
HPV 31	26	(23–28)	1,554	(1,416–1,704)	176	(151–205)	88	(76–102)
HPV 33	36	(32–39)	2,540	(2,275–2,836)	274	(230–326)	123	(103–148)
HPV 45	8	(7–9)	462	(408–522)	45	(37–54)	25	(21–30)
HPV 52	96	(85–109)	2,960	(2,686–3,261)	368	(314–431)	184	(158–214)
HPV 58	93	(85–102)	3,483	(3,162–3,838)	435	(375–504)	207	(176–245)

^a^international units (IU)/mL

GMC at three years after vaccination stratified by sex and age at enrollment are presented in Table 4. GMC were higher among females compared to males for four of the 9vHPV types (HPV 16, 31, 33, 45) at three years post dose two. The association between higher GMC and female sex remained statistically significant for HPV 16, 31, 33, and 45 in linear regression models after controlling for age (*p* < 0.03 for all four types).

**Table 4:** Geometric mean concentrations (GMC) and 95% confidence intervals (CI) for type-specific human papillomavirus (HPV) immunoglobulin G antibodies at three years after completion of a two-dose series of nine-valent HPV vaccine among Alaska Native children, by sex and age group at first dose

**Type**	**Males** **[*N* = 79]**		**Females** **[*N* = 66]**			**9–11 years old** **[*N* = 116]**		**12–14 years old** **[*N* = 29]**	
	GMC^a^	(95% CI)	GMC^a^	(95% CI)	*p*-value^b^	GMC^a^	(95% CI)	GMC^a^	(95% CI)
HPV 6	186	(145–240)	267	(207–344)	0.05	226	(185–276)	195	(127–300)
HPV 11	154	(123–193)	193	(157–238)	0.15	178	(149–213)	147	(108–199)
HPV 16	80	(64–100)	115	(93–143)	0.02	101	(85–119)	73	(47–113)
HPV 18	24	(18–31)	32	(25–40)	0.11	28	(23–34)	23	(15–37)
HPV 31	74	(61–91)	108	(88–132)	0.01	95	(82–110)	65	(44–97)
HPV 33	103	(80–131)	154	(119–199)	0.03	129	(105–158)	103	(68–156)
HPV 45	21	(16–27)	31	(24–40)	0.03	26	(22–32)	21	(13–33)
HPV 52	161	(132–197)	215	(170–270)	0.07	196	(166–230)	143	(96–211)
HPV 58	183	(143–234)	241	(194–299)	0.10	218	(181–263)	171	(119–245)

^a^international units (IU)/mL

^b^independent t-test assuming unequal variances

## Discussion

In this prospective, longitudinal study of the immunogenicity of a two-dose series of 9vHPV among Alaska Native children, all participants were seropositive for antibodies against all nine HPV types at one month after series completion and 92% remained seropositive for all types at three years after vaccination. Anti-HPV IgG GMC peaked one month following dose two and remained higher at three years compared to GMC six months post dose one. Previously published studies of immunogenicity following receipt of a two-dose series of 9vHPV were among cohorts from randomized controlled trials and report results up to 2.5 years post dose two [10–12]. We report duration of antibodies at three years after a two-dose 9vHPV series among children vaccinated under real-world conditions during routine clinical care. Our findings are consistent with studies evaluating the immunogenicity of a two-dose series of 4vHPV and 9vHPV and show that the kinetics of antibody response are similar after a two- and three-dose series [8–12, 15, 19–22].

At six months after vaccination with one dose of 9vHPV, anti-HPV IgG seropositivity was ≥ 94% for eight of nine types. However, only 85% of participants remained seropositive for antibodies against HPV 45. In a randomized controlled trial that evaluated immune response 12 months after one dose of 9vHPV, seropositivity also was lowest against HPV 45 [11]. While the clinical relevance of this observation is unknown, an evaluation of the long-term immunogenicity and effectiveness of a three-dose series of 9vHPV suggests that children who receive additional doses of 9vHPV are likely protected from disease due to HPV 45 despite potentially lower post-dose one seropositivity for that type [9].

At three years post vaccination, anti-HPV IgG GMC had decreased by 94–96% from peak levels for all 9vHPV types, and seropositivity was lowest for HPV 18 and HPV 45. Previous studies also have reported lower seropositivity for HPV 18 and HPV 45, although the reason for this finding is unclear [23, 24]. Strong anamnestic responses following additional doses of HPV vaccine have been observed for all 9vHPV types, including HPV 18 and HPV 45, suggesting humoral immune memory [23–25]. Evaluations demonstrating long-term effectiveness against vaccine-type disease despite antibody decay provide further evidence of immune memory and suggest that waning anti-HPV IgG does not indicate waning protection from the serious sequelae of HPV infection [18, 26].

We observed higher antibody levels in females compared to males at three years after vaccination for four 9vHPV types. Sex-based differences in immune response to vaccination have been reported for several viral vaccines and a variety of mechanisms have been hypothesized to explain these findings [27]. A recent meta-analysis of 4vHPV immunogenicity studies found that females had higher antibody levels than males after vaccination, regardless of age [28]. However, a previous study among Alaska Native children found no difference in IgG response to 4vHPV between the sexes after controlling for age [15]. A post hoc analysis of data from two 9vHPV clinical trials in children aged 9–15 years found higher antibody levels in females compared to males after one dose of 9vHPV, similar levels between the sexes after two doses, and higher levels in males after three doses [29]. Questions remain about potential sex-based differences in immune response after vaccination with 9vHPV. Importantly, long-term follow-up studies of HPV vaccine effectiveness have demonstrated persistent protection for both males and females [8, 9].

Historically, HPV vaccine coverage among Alaska Native children has been higher than among U.S. children overall [30]. However, the proportion of Alaska Native children up to date with HPV vaccination during 2017–2019 was 62%, which is below the Healthy People 2030 goal of 80% [30, 31]. In a study of Alaska Native adolescent views on HPV and HPV vaccine, participants identified concerns about vaccine efficacy and side effects as barriers to vaccination [32]. Communication of the robust data on safety and effectiveness of HPV vaccines to clinicians and patients might help address these concerns [33].

Our study has several limitations. First, we did not collect a pre-vaccination blood sample and were unable to determine if participants were seropositive for anti-HPV IgG before receiving HPV vaccine. Participants who were seropositive at baseline might have an enhanced immune response to 9vHPV, which could cause overestimation of post-vaccination anti-HPV seropositivity and GMC. Second, an immunologic correlate of protection has not been established for HPV and it is unknown if the antibody levels we observed confer protection from HPV infection. Third, the small number of participants in the cohort aged 12–14 years prevented us from testing for an association between age group and immune response to vaccination. Finally, some participants may have been naturally exposed to HPV during the study period, which could have impacted the antibody levels we observed.

## Conclusions

A two-dose series of 9vHPV was highly immunogenic in this cohort of Alaska Native children. More than 90% of participants remained seropositive for all 9vHPV types at three years after vaccination. Our findings support the recommendation that all children, including Alaska Native children, receive HPV vaccine according to ACIP guidelines. Follow-up for this study will continue through 20 years post vaccination to determine duration of antibody detection in this cohort.

## Supplementary Information

Supplementary Material 1.

## Data Availability

The datasets generated and/or analyzed during the current study are not publicly available as they were collected from children under a research protocol and are considered sensitive. Access to anonymized data will be considered on reasonable request and following approval by the Alaska Area Institutional Review Board, Alaska Native Tribal Health Consortium, Southcentral Foundation, and the Centers for Disease Control and Prevention.

## Abbreviations

9vHPV: 9-Valent human papillomavirus vaccine
ACIP: Advisory Committee on Immunization Practices
CDC: Centers for Disease Control and Prevention
GMC: Geometric mean concentration
HPV: Human papillomavirus
IgG: Immunoglobulin G
IU: International units
4vHPV: Quadrivalent human papillomavirus vaccine
