# Supplementary material for: Immunogenicity and duration of antibodies after vaccination with a two-dose series of the nine-valent human papillomavirus vaccine among Alaska Native children: a prospective cohort study
Source: BMC Infect Dis. 2025 Apr 30;25:640. doi: 10.1186/s12879-025-10961-z (PMC12044979; doi:10.1186/s12879-025-10961-z)
Supplement: Supplementary file 1 — Supplementary Material 1. [file 12879_2025_10961_MOESM1_ESM.docx]

**9vHPV Immunogenicity Study**

**Eligibility Screening Form**

9–14 years of age? □Yes □No

Resides in the Anchorage urban area (Anchorage, Eagle River, Palmer, Wasilla)? □Yes □No

Is eligible for care at Southcentral Foundation clinics? □Yes □No

Prior receipt of QHPV vaccine? □Yes □No

Hypersensitivity to the components of the vaccine (yeast)? □Yes □No

Plans to leave the Anchorage area within 6 months of entry into the study? □Yes □No

Existence of immune deficiency (e.g., HIV, AIDS, SCID, chronic renal disease □Yes □No

requiring dialysis, nephrotic syndrome, leukemia, lymphoma, Hodgkin’s disease,

multiple myeloma, organ or bone marrow transplant, generalized malignancy)?

Current or recent (within 6 months) receipt of immunomodulatory therapy □Yes □No

(e.g., systemic corticosteroids, chemotherapy) or blood products?

Current pregnancy (self-reported)? □Yes □No
